# Supplementary material for: Poliovirus-Neutralizing Antibody Seroprevalence and Vaccine Habits in a Vaccine-Derived Poliovirus Outbreak Region in the Democratic Republic of Congo in 2018: The Impact on the Global Eradication Initiative
Source: Vaccines (Basel). 2024 Feb 27;12(3):246. doi: 10.3390/vaccines12030246 (PMC10975469; doi:10.3390/vaccines12030246)
Supplement: Supplementary file 1 [file vaccines-12-00246-s001.zip › vaccines-2839741-supplementary.pdf]

Table S1. Participant demographics by poliovirus seroprofile

	Study pop		None		Type 1		Type 2		Type 3		Type 2 Only		All	
	**n**	**Col %**	**n**	**Col %**	**n**	**Col %**	**n**	**Col %**	**n**	**Col %**	**n**	**Col %**	**n**	**Col %**
**Polio Seroprevalenace**														
Type 1	422	43.78	.	.	422	100	260	65.66	279	76.23	.	.	172	100
Type 2	396	41.08	.	.	260	61.61	396	100	204	55.74	104	100	172	100
Type 3	366	37.97	.	.	279	66.11	204	51.52	366	100	.	.	172	100
**Age**														
6 to 11 months	315	32.68	146	41.6	109	25.83	106	26.77	94	25.68	40	38.46	41	23.84
12 to 23 months	609	63.17	191	54.42	294	69.67	268	67.68	254	69.4	62	59.62	118	68.6
24 to 35 months	38	3.94	12	3.42	19	4.5	22	5.56	18	4.92	2	1.92	13	7.56
**Province**														
Tanganyika	637	66.08	252	71.79	283	67.06	230	58.08	243	66.39	55	52.88	113	65.7
Haut Lomami	327	33.92	99	28.21	139	32.94	166	41.92	123	33.61	49	47.12	59	34.3
**Health Zone SIA History**														
4-5 SIAs: Haut Lomami	327	33.92	99	28.37	139	32.94	166	41.92	123	33.61	49	47.12	59	34.3
2 SIAs: Ankoro & Manono	331	34.34	111	31.81	146	34.6	171	43.18	128	34.97	46	44.23	82	47.67
0 SIAs: Kabalo & Kongolo	306	31.74	141	40.40	137	32.46	59	14.9	115	31.42	9	8.65	31	18.02
**Sex**														
Male	509	52.8	178	50.71	236	55.92	208	52.53	193	52.73	55	52.88	92	53.49
Female	455	47.2	173	49.29	186	44.08	188	47.47	173	47.27	49	47.12	80	46.51
**Vaccine Card Present**														
Yes	126	13.07	31	8.83	69	16.35	61	15.4	71	19.4	9	8.65	37	21.51
No	554	57.47	169	48.15	278	65.88	247	62.37	225	61.48	58	55.77	106	61.63
Missing	284	29.46	151	43.02	75	17.77	88	22.22	70	19.13	37	35.58	29	16.86
**Educational Achievement**														
None	190	19.71	70	19.94	68	16.11	83	20.96	69	18.85	33	31.73	31	18.02
Primary school or appretice	551	57.16	201	57.26	237	56.16	216	54.55	207	56.56	59	56.73	92	53.49
Finished secondary school	209	21.68	74	21.08	111	26.3	90	22.73	86	23.5	10	9.62	45	26.16
Higher education	10	1.04	4	1.14	5	1.18	6	1.52	4	1.09	1	0.96	4	2.33
**Transportation to Health Facility**														
Walk	908	94.19	332	94.59	402	95.26	369	93.18	350	95.63	92	88.46	164	95.35
Boat/Pirogue	22	2.28	9	2.56	8	1.9	12	3.03	4	1.09	5	4.81	4	2.33

Bicycle	21	2.18	4	1.14	7	1.66	11	2.78	8	2.19	6	5.77	2	1.16
Moto	13	1.35	6	1.71	5	1.18	4	1.01	4	1.09	1	0.96	2	1.16
**Time to Health Facility**														
<10 minutes	376	39	129	36.75	172	40.76	169	42.68	149	40.71	40	38.46	80	46.51
11 to 30 min	308	31.95	120	34.19	125	29.62	119	30.05	119	32.51	32	30.77	52	30.23
30 to 1 hour	135	14	45	12.82	62	14.69	56	14.14	51	13.93	17	16.35	23	13.37
> 1 hour	141	14.63	56	15.95	61	14.45	49	12.37	46	12.57	14	13.46	16	9.3
Dont Know	4	0.41	1	0.28	2	0.47	3	0.76	1	0.27	1	0.96	1	0.58
**Number of Children Under 5**														
0	10	1.04	3	0.85	6	1.42	5	1.26	6	1.64	.	.	4	2.33
1	185	19.19	64	18.23	83	19.67	78	19.7	77	21.04	23	22.12	40	23.26
2-3	598	62.03	213	60.68	260	61.61	249	62.88	229	62.57	67	64.42	106	61.63
4-5	128	13.28	57	16.24	51	12.09	45	11.36	39	10.66	10	9.62	17	9.88
6+	43	4.46	14	3.99	22	5.21	19	4.8	15	4.1	4	3.85	5	2.91
